# *Listeria monocytogenes* sensitivity to antimicrobial treatments depends on cell origin

**DOI:** 10.1038/s41598-021-00767-9

**Published:** 2021-10-28

**Authors:** Chiara Montanari, Giulia Tabanelli, Federica Barbieri, Diego Mora, Robin Duncan, Fausto Gardini, Stefania Arioli

**Affiliations:** 1 Department of Agricultural and Food Sciences, University of Bologna, Bologna, Italy; 2 Interdepartmental Center for Industrial Agri-Food Research, University of Bologna, Bologna, Italy; 3 Department of Food, Environmental and Nutritional Sciences (DeFENS), Università Degli Studi di Milano, Via Celoria 2, 20133 Milan, Italy

**Keywords:** Antimicrobials, Pathogens

## Abstract

In this study we investigated how cell origin could affect the efficacy of an antimicrobial treatment (mild heating combined with terpenoids) in *Listeria monocytogenes* Scott A, considering cells from: 1. single colony, 2. glycerol stock, 3. cold adapted culture, and 4. fresh culture in stationary phase. After treatment, culturability on BHI medium and viability assessed by flow cytometry were evaluated. Our results showed that the cell origin significantly impacted viability and culturability of *L. monocytogenes* towards antimicrobial treatment. The mild heat treatment combined or not with terpenoids mainly affected culturability rather than viability, although the culturability of cells from single colony was less impacted. Therefore, to mimic the worst scenario, these latter were selected to contaminate Gorgonzola rind and roast beef slices and we evaluated the ability of *L. monocytogenes* cells to recover their culturability (on ALOA agar medium) and to growth on the food matrix stored at 4 °C for 7 days. Our results suggest that only Gorgonzola rind allowed a partial recovery of the culturability of cells previously heated in presence or not of terpens. In conclusion, we found a connection between the cell history and sensitivity toward an antimicrobial treatment, underlying the importance to standardize the experimental procedures (starting from the cells to be used in the assay) in the assessment of cell sensitivity to a specific treatment. Finally, our study clearly indicated that VBNC cells can resuscitate under favorable conditions on a food matrix, becoming a threat for consumer’s health.

## Introduction

*Listeria monocytogenes* is a ubiquitous bacterium responsible for a disease (listeriosis) which is among the major foodborne illnesses characterized by a high case-fatality rate (approximately from 20 to 30%). Its wide environmental distribution, resistance to low pH and NaCl concentration, facultatively anaerobic metabolism and psychrotrophicity make this species one of the greater challenges for food industry^1^. Due to these characteristics, the risk of listeriosis is high in ready-to-eat foods (meat and dairy products) but relevant outbreaks has been associated also to fruit and vegetables (cantaloupe, celery, mung bean sprouts, stone fruits)^2^. In many cases, the control of this microorganism relies on thermal processing of foods. Temperatures above 50 °C are needed for thermal death of *L. monocytogenes* cells. However, the inactivation kinetics can be very different, with D_65_ ranging from 0.2 to 2 min according to ANSES (2012)^3^. Recently, revision of literature confirmed this variability in D-value^4^, indicating that it may change in relation to temperature suggesting non-linear inactivation dynamics^5^. On the other hand, the application of high temperature may have negative effect on the sensory, nutritional, and textural characteristics of food that may be contaminated by *L. monocytogenes*. Therefor strategies based on synergistic effects of thermal treatment with antimicrobials are exploited to avoid excessive heat damages. In this perspective, the use of essential oils or their constituents (added below their minimum inhibitory concentration) can be a suitable approach: the presence of thymol, carvacrol, citral, *(E)*-2-hexenal, vanillin is reported to increase the inactivation rate of thermal treatments^6–11^, due to cumulative antimicrobial effects of heat and aroma compounds. These latter are enhanced by the increase of vapour pressure caused by heat which, in turn, increases their solubility in cell membrane, the first target of their antimicrobial activity^12,13^. Inactivation results obtained in heat treatment experiments are subjected to a variability which depends on several factors. In addition to the process parameters, a relevant part of this variability is associated to the different responses of the treated cells. Variation can characterize strains of different species, strains of the same species or the same microorganism grown under different environmental conditions^14^.

Laboratory tests on stress responses of microorganisms of concern in foods, such as thermal treated *L. monocytogenes,* are often carried out using liquid batch cultures, which are assumed to be phenotypically homogeneous. This assumption has recently been debated by Kragh et al.^15^ which demonstrated the relationship between the method of inoculation of liquid batch cultures (*Pseudomonas aeruginosa*) and antibiotic tolerance (tobramycin). The effect of the physiological state of a broth culture (cells in exponential phase or in stationary phase) has already been discussed^16^ being cells in exponential phase more susceptible to heat^17^. However, Kragh et al.^15^ posed their attention on the history of the cells used for the preparation of the liquid inoculum and observed that second generation cultures (obtained even after a 1000-fold dilution) were able to maintain phenotypic characteristics deriving from the status of the initial pre-cultures (colony, liquid, frozen stock) keeping a sort of memory, mainly related to exopolysaccharides production and ability to form aggregates. Other studies reported the influence of the age and conditions of inoculum on the frequency *Escherichia coli* cells able to survive antibiotic treatment^18^. In *Staphylococcus aureus* the growth mode and the growth stage strongly influenced the surfaceome, and consequently the cell sensitivity to levofloxacin^19^. In *L. monocytogenes* the effects of the growth temperature and growth phase on the cell inactivation after high pressure processing in milk were evaluated^20^.

In the current work, cells of *L. monocytogenes* of 4 different origins were subjected to a mild thermal treatment (55 °C) in the presence or in the absence of two terpenoids with antimicrobial activity (thymol and carvacrol). The effect of the treatment was evaluated by monitoring the culturability (by plate counting) and the viability (by flow cytometry) of the treated cells. Then, the growth performances of the survived cells were modeled with the Gompertz equation. The variation of the resulting parameter *λ* (lag phase duration) was further analysed for evaluating the variability of the responses in relation to the treatment and to the cell history. The cells deriving from the condition able to guarantee the higher recovery after heat treatments were chosen to perform a trial in food systems, namely Gorgonzola rind and roast beef slices.

## Results and discussion

### Effects of treatments on viability and culturability of *L. monocytogenes*

**Figure 1:**
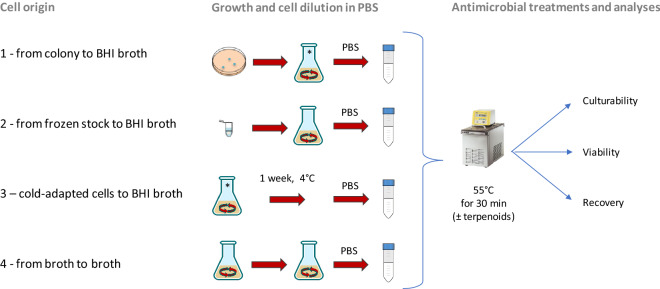
Schematic representation of four cell origin used in this study. Origin 1: from single colony to BHI broth; origin 2: from frozen stock to BHI broth; origin 3: cells derived from the condition 1 and then cold-adapted (1 week at 4 °C); origin 4: from culture in early stationary phase to BHI broth.

To examine the impact of cell origin on the efficacy of mild heat treatment combined or not with terpenoids, *L. monocytogenes* cells were cultivated in different conditions (Fig. 1). After growth, cells diluted in PBS were subjected to the antimicrobial treatment, in presence or not of terpenoids at sublethal concentrations, based on the previous determination of minimum inhibitory concentration (MIC)^6^. The results obtained are summarized in Table 1. In control cells no significative differences in terms of Active Fluorescent Unit (AFU)/ml and CFU/ml (viability and culturability, respectively) were observed, confirming an initial inoculum of approx. 6 log AFU or CFU/ml.

**Table 1:** Viability, culturability, and recovery in BHI broth of *L. monocytogenes* cells after mild heat treatment combined with terpens.

Cell origin	Control (untreated cells)						55 °C						55 °C TC					
	Plate counting log CFU/ml	Flow cytometry^a^				Growth 37°C^b^ %	Plate counting log CFU/ml	Flow cytometry				Growth 37°C^b^ %	Plate counting log CFU/ml	Flow cytometry				Growth 37°C^b^ %
		log AFU/ml^c^	Alive %	Injured %	Dead %			log AFU/ml	Alive %	Injured %	Dead %			log AFU/ml	Alive %	Injured %	Dead %	
1	6.06a (± 0.24)	5.99a (± 0.07)	97.42	1.22	1.36	100	4.12b (± 0.35)	5.96a (± 0.05)	93.10	1.84	5.06	100	3.08c (± 0.42)	5.84a (± 0.22)	75.79	6.96	17.25	100
2	6.03a (± 0.07)	6.11a (± 0.07)	98.52	1.06	0.42	100	2.89b (± 0.37)	5.84a (± 0.19)	76.69	5.87	17.43	79	< 1	4.77c (± 1.07)	18.33	18.05	63.62	0
3	6.08a (± 0.16)	5.89a (± 0.27)	96.70	1.11	2.18	100	3.42b (± 0.33)	5.78a (± 0.21)	78.92	5.08	16.00	100	1.97c (± 0.67)	5.16d (± 0.40)	33.52	14.82	51.66	43
4	6.07a (± 0.25)	5.94a (± 0.07)	98.29	1.07	0.65	100	3.50b (± 0.49)	5.84a (± 0.10)	95.63	1.76	2.64	50	< 1	5.58c (± 0.18)	83.83	7.79	8.39	11

^a^Data are reported as total cells counted clustered as AFU (considered as alive cells), injured and non-AFU (considered as dead cells), expressed as percentages of the total cell number. For each inoculum, significant differences in culturability and viability between samples according to ANOVA are indicated by lowercase letters.

^b^Expressed as percentage of growth after the different treatments in the 28 repetitions for each condition.

Based on flow cytometry data in non-treated cell suspensions we did not highlight relevant differences in terms of distribution of the 3 subpopulations (AFU, damaged and non-Active Fluorescent Unit non-AFU), indicating that the cell origin did not affect the viability of the cell, even after one week of storage at 4 °C. After the thermal treatment at 55 °C, the viability was not significantly affected in all tested condition. By contrast, the culturability was severely compromised. In fact, a decrease of culturability of 3.14, 2.66 and 2.57 log cfu/ml was observed for cell origin 2, 3 and 4, respectively (Table 1). Conversely, cells derived from condition 1 (from colony) were the less affected by the thermal treatment, with a decrease of culturability of about 1.90 log cfu/ml. The thermal treatment in presence of thymol and carvacrol increased the discrepancy between viability and culturability. Indeed, the viability decreased from 0.36 up to 1.34 log AFU/ml for cells from origin 2, 3 and 4. Conversely, cells from condition 1 was not significantly affected by the treatment. In parallel, the culturability was almost or completely abolished. Indeed, the culturability of cold-adapted cells (condition 3) was about 1.97 log cfu/ml and below the detection limit (< 1 log CFU/ml) in conditions 2 and 4. Conversely, the culturability of cells derived from colony to broth (condition 1) was less affected compared to the other conditions (3.08 log CFU/ml).

The synergistic effect between mild thermal treatment and antimicrobials has already been demonstrated for several microorganisms and conditions. Concerning *L. monocytogenes*, a relevant reduction of the treatment time required to obtain the same cell inactivation has already been reported in the presence of thymol, carvacrol, citral and *(E)*-2-hexenal^6,11^ and the synergy between essential oils and thermal treatment on foodborne pathogens has been recently reviewed by Gurtler et al.^12^. In first instance, the results obtained in these trials demonstrated that *L. monocytogenes* cells grown in aerobic conditions (stirred in a flask at 150 rpm) had reduced susceptibility than those grown in static conditions^6^, underlying the importance of the cell cultivation on the effectiveness of antimicrobial treatments. On the other hand, relevant differences in growth and survival performances of *L. monocytogenes* in relation to presence/absence of oxygen are well known^21,22^. In addition, as observed by Kragh et al.^15^, it is often assumed that a liquid batch culture contains phenotypically homogenous cells. However, these authors demonstrated that the inoculation method (i.e. cell history) has a relevant effect on the phenotypes of the resulting population. They demonstrated also that some characteristics (in the specific case the frequency of aggregation of *Pseudomonas aeruginosa*) of the cultures first seeded can be inherited in second generation cultures modifying responses to stress such as the presence of antibiotics.

In any case, the discrepancies between viability and culturability expressed as log CFU/ml raise the question of the viable but not culturable cells (VBNC) that in some cases consist in several log cells/ml. As stressed by Donnelly and Diez-Gonzalez^23^, improvement in testing methods for *L. monocytogenes* are needed to ensure an adequate sensitivity of detection for identifying and control the presence of this pathogen. Particularly, a better understanding of factors affecting the recovery and growth of VBNC cells is needed to determine the real effect of an antimicrobial treatment. Indeed, the VBNC state may be reversible (“resuscitation”) and this condition is a concern when it involves storage of food material^23^.

Studies regarding the efficacy of inactivation methods on *L. monocytogenes* reported similar results that raised the same issues. For example, Alessandria et al.^24^ observed that after a treatment with cold atmospheric pressure plasma (APP) most of the culturable cells were inactivated but, when inoculated in BHI medium (therefore removing the environmental stress), some of them were able to regrow.

Although it is still matter of discussion if this regrowth is the result of a true resuscitation phenomenon or it is rather due to few residual culturable cells^25^, these observations highlight the need to integrate culture-dependent and culture-independent approaches to monitor this pathogen, since conventional culture methods can overestimate the efficacy of the treatments performed.

The same considerations were reported by Noll et al.^26^, whose study evidenced that *L. monocytogenes* adapted to antimicrobial agents (in that case benzalkonium chloride) were viable and metabolically active according to FCM analyses but not detectable by standard cultivation techniques, indicating the occurrence of VBNC cells. Gu et al.^27^ investigated the susceptibility of some foodborne pathogens (including *L. monocytogenes*) to sanitizers such as free chlorine and peracetic acid. They hypothesized the induction of a VBNC state, already reported in literature for these chemicals^28^, because of the discrepancy between the inability to detect cells by plating and the results of PMA-qPCR and laser confocal microscopy, that evidenced large amounts of DNA from theoretically viable bacterial cells. However, the conditions adopted in that study did not allow resuscitation of this population in vitro, but a possible recovery in vivo cannot be excluded, raising alarming threats for food safety.

### Effects of antimicrobial treatments and cell origin on recovery ability and growth kinetics

To test whether the cell origin could influence the ability of the cells to recover after antimicrobial treatment, stressed cells were inoculated in a 96-well plate (for a total of 28 wells/repetitions for each condition) for 48 h at 37 °C. The recovery was expressed as percentage of wells displaying growth on the total of inoculated wells (Table 1). As expected, 100% of the wells inoculated with non-treated cells of *L. monocytogenes* displayed growth, regardless of the cell origin. Concerning the ability to recover after the treatment at 55 °C, all cells were able to recover but to different extent. Only cells from condition 1 and 3 displayed growth in 100% of the inoculated wells. Conversely, less efficient was the recovery of cells from condition 2 and 4, with percentages assessed at 79 and 50%, respectively. When cells were subjected to the thermal treatment combined with terpenoids, the recovery capability was strongly impacted with an evident influence of the cell history. Cells from condition 2 were not able to recover after treatment. Conversely, the recovery of cells from conditions 3 and 4 was 43 and 11%, respectively. Instead, all wells inoculated with cells derived from condition 1 displayed cells growth. These differences can be explained by the culturability of treated cells (expressed as log cfu/ml). In fact, in the sample treated at 55 °C, the dilution used for the inoculation of wells (1:100) determined a mean inoculum of culturable cells of about 1 log cfu/ml or even less (e.g. the case of cells from condition 2 after treatment at 55 °C; all cases after the mild heat treatment combined with terpenoids). The variability associated with these low inoculums could explain the presence of wells with no visible growth. By contrast, considering the sample thermally treated in the presence of terpenoids, the growth percentage did not reflect the associated culturability. The growth observed could be due to unculturable cells which however resulted alive according to the FCM protocol. This consideration opens the question of the fate of these cells over time.

**Figure 2:**
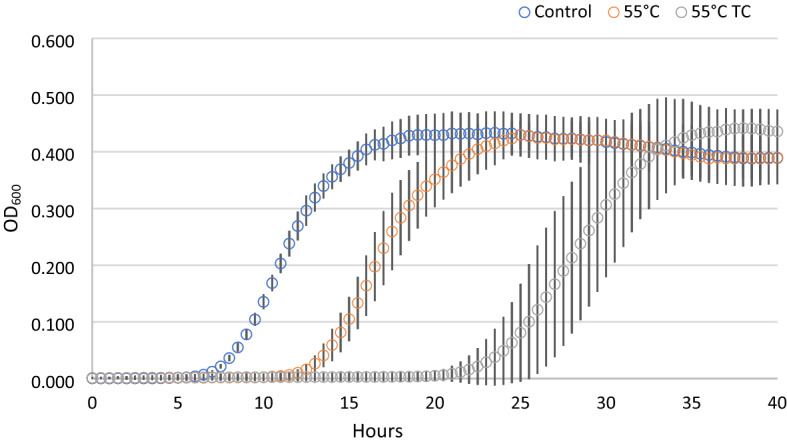
Growth curves of cells derived from condition 1, after antimicrobial treatments. Each time point is the mean of 28 replicates. For each point standard deviation is reported.

**Figure 3:**
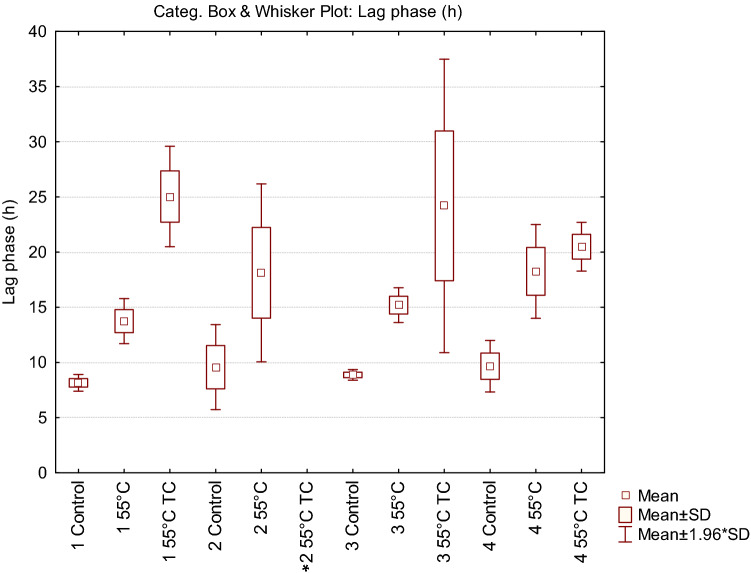
Box and Whisker plot describing the variability of lag phase (λ) in relation to the different cell origin and treatment. In the condition marked by an asterisk no growth was observed in any of the 28 replicates.

In parallel to the recovery ability, we investigated the kinetic parameters of the treated cells deriving from condition 1, compared with untreated control cells (Fig. 2). Each point of the growth curves represented the mean of the observation recorded in 28 experimental replicates. It is evident the delay in the growth curves induced by the thermal treatment. Even more noticeable was the effect on the lag phase lasting of the thermal-treated cells in presence of the two terpenoids. In addition, the analysis of the standard deviation of all the reading points of the growth curves showed how the variability was dependent on the kind of antimicrobial treatment (55 °C TC > 55 °C > Untreated). The data concerning the samples in which growth was observed were then modeled with the Gompertz equation, as modified by Zwietering et al.^29^. The measurement of the OD is an indirect method in which the absorbance changes occurs when the bacterial concentration reaches a minimum bacterial concentration of approx. 7 log cells/ml, after which the OD linearly increases up to a concentration of 8 log cells/ml reaching the higher absorbance allowed by the instrumental conditions^30^. The parameter *λ* of the model (lag phase duration) was used to estimate the time needed to obtain this critical threshold, i.e. necessary to have an increase of the OD_600_ baseline. Figure 3 reports the Box and Whisker plots regarding the *λ* values estimated in the wells in which growth occurred. Independently of the inoculum, *λ* increased, as expected, with the severity of the treatment. Noteworthy, also the variability of the responses increased with the stress applied. This variability was particularly relevant in the thermal treatment in the presence of terpenes, with the exception of inoculum 4 (inoculum from a liquid culture to fresh medium); however, in this latter case the data are affected by the presence of only 11% (3 out of 28) of positive cases. The lack of homoscedasticity of the variance did not allow the use of ANOVA to exploit the difference among the samples. Even after a log transformation of *λ* the requisite was not accomplished (data not shown). For this reason, the non-parametric Kruskal–Wallis test^31^ was applied and the results are reported in Table 2. The result of this test showed a significant difference between the ranks of *λ* and the treatments (P < 0.001). Untreated samples were not significantly different with the exception of inoculum 1 (direct inoculation from colony to broth) which showed lower *λ* values. Among the thermal treated samples, no differences were observed between inoculum 2 (direct inoculation from frozen stock) and 4 (inoculum from early stationary phase culture to fresh medium), which, however, showed respectively 79 and 50% of growth among the inoculated samples. Inoculum 1 and 3 (cold adapted cells), in which all the replicates grew, showed significantly lower *λ* values. The samples added with thymol and carvacrol were grouped together, even if the samples from inoculum 4 did not present significant differences with the samples obtained from inoculum 2 and inoculum 4 thermally treated at 55 °C. Nevertheless, in this latter case, only in 3 out of 28 samples the growth was observed.

**Table 2:** Results of the application of the non-parametric Kruskal Wallis test to the lag phase (*λ*) estimates in the different conditions.

Cell origin	Treatment	Mean^a^	SD^b^	N^c^	Min^d^	Max^e^	Q25^f^	Q50^g^	Q75^h^	Groups^i^
1	Control	8.16	0.38	28	7.27	8.69	7.84	8.36	8.41	a
2	Control	9.58	1.96	28	7.53	11.73	7.70	9.31	11.60	b
3	Control	8.88	0.25	28	8.32	9.34	8.68	8.92	9.07	b
4	Control	9.66	1.16	28	7.78	10.93	8.56	10.19	10.77	b
1	55 °C	13.75	1.04	28	12.11	17.16	13.16	13.62	14.10	c
2	55 °C	18.13	4.11	22	13.34	29.25	14.91	17.20	20.82	d
3	55 °C	15.20	0.81	28	13.22	16.41	14.62	15,023	15.88	e
4	55 °C	18.25	2.17	14	11.78	20.84	17.76	18.64	19.46	d
1	55 °C TC	25.05	2.32	28	20.97	30.50	23.68	24.92	26.23	f
2	55 °C TC^e^	–	–	0	–	–	–	–	–	–
3	55 °C TC	24.19	6.79	12	15.90	37.81	20.30	20.96	27.53	f
4	55 °C TC	20.50	1.13	3	19.21	21.30	20.09	20.98	21.14	fd

^a^Mean value for *λ.*

^b^Standard Deviation.

^c^Number of replicates in which growth was observed (out of 28 replicates).

^d^Minimum *λ* value observed.

^e^Maximum *λ* value observed.

^f^25th quartile.

^g^50th quartile (median).

^h^75th quartile.

^i^Grouping of the treatments according to Kruskal Wallis test (p < 0.01).

### Recovery of *L. monocytogenes* cells in food model systems

**Figure 4:**
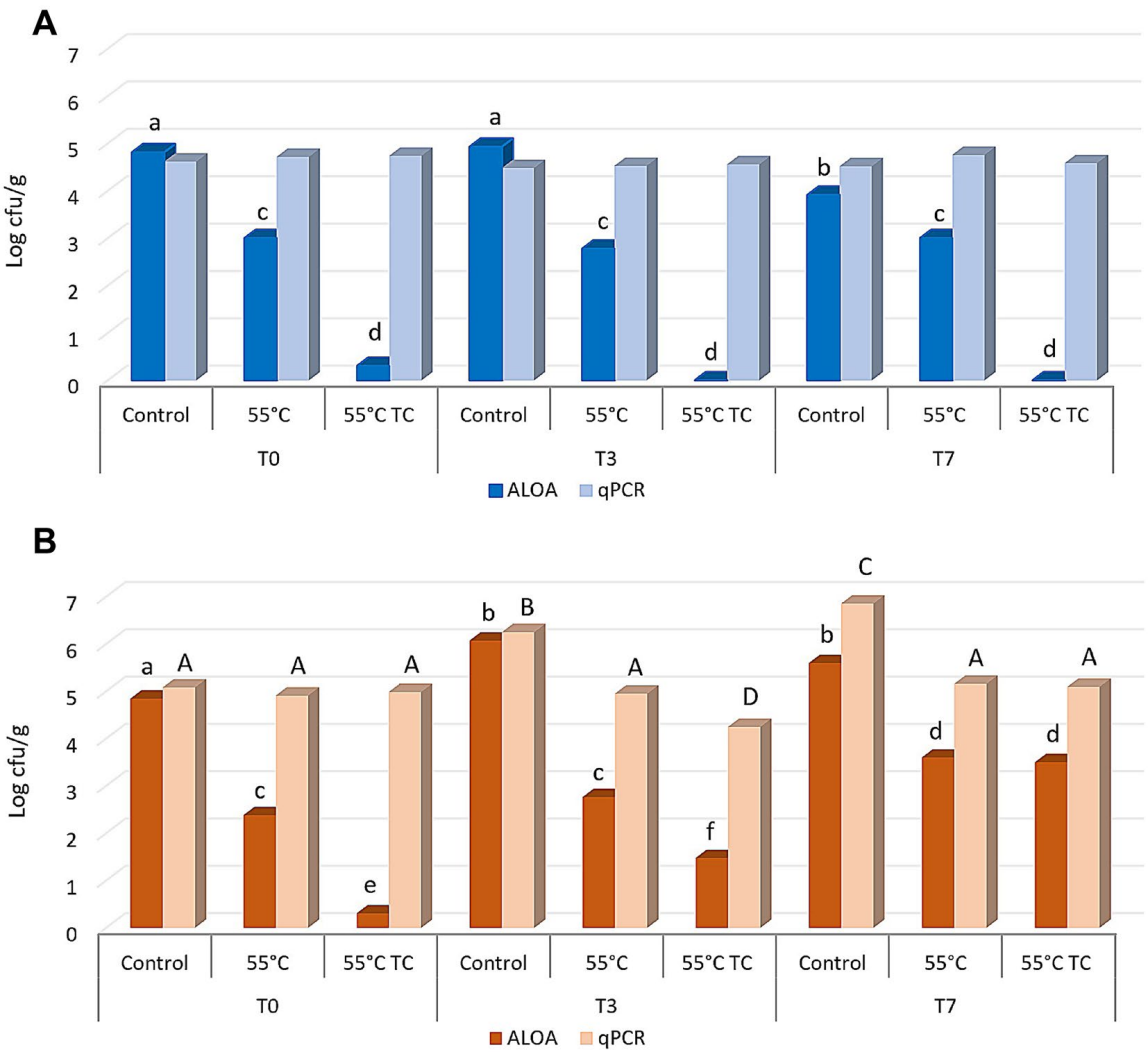
Culturability on ALOA and total *L. monocytogenes* cell counting by qPCR with species specific primer on roast beef slices (**A**) and Gorgonzola rind (**B**) stored at 4 °C after contamination. Before the inoculum cells were not treated (Control), treated at 55 °C (55 °C) or treated at 55 °C in the presence of thymol and carvacrol (55 °C TC). Significant differences between samples according to ANOVA are indicated by lowercase letters for cell culturability and capital letters for cell counting by qPCR.

Based on viability, culturability and recovery data, the cells deriving from inoculum 1 (direct inoculation of a colony in liquid medium and growth for 24 h) resulted the more prompted to counteract the applied stress. To mimic the worst scenario, these cells were used to contaminate 2 different food matrices. The attention was focused on Gorgonzola cheese and sliced roast beef, which can be susceptible to *L. monocytogenes* colonization^32,33^. Both matrices were inoculated with non-treated (control) and treated cells (55 °C and 55 °C TC). The size of inoculum was around 4.6–4.8 log cell/g (50 µl of the original cell suspension in PBS) and after inoculation the samples were stored at 4 °C, then analysed after 3 and 7 days of incubation. Microbiological analyses were carried out using plate counting as well as qPCR to determine culturability and the total *L. monocytogenes* cell counting, respectively. A non-inoculated sample was also considered to exclude the presence of *L. monocytogenes* in the raw materials. The results indicated that this pathogen was not detected, neither by cultivation nor molecular methods, in non-inoculated samples for both kind of foods (data not shown). Results concerning roast beef slices (Fig. 4A) showed that at T0 the counting in the control sample was 4.81 log CFU/g (according to plate counting) and 4.61 log cells/g (according to q-PCR). In the treated samples, the qPCR confirmed the same cell concentration, but the culturability decreased at 3.0 log CFU/g in the thermal treated cells. The presence of terpenoids determined a higher decrease of the culturability (below 1 log CFU/g) since T0. However, treated cells were not able to recover their culturability after 3 and 7 days of incubation. Moreover, the total number of *L. monocytogenes* cells (qPCR data) remained stable over the time and in all samples, including control cells. Our results are in accordance with Skjerdal et al.^34^, where authors did not find any growth when *L. monocytogenes* was inoculated in a challenge test on roast beef slices, confirming that roast beef is not an ideal substrate for the growth of the cells when stored at refrigerated temperatures.

A different scenario was observed when cells were inoculated on Gorgonzola rind (Fig. 4B). The initial quantifications at T0 were similar to those obtained for roast beef, both in terms of log CFU/g and total number of cells/g. Conversely to roast beef, control cells of *L. monocytogenes* were able to increase the total cell concentration after 3 days at 4 °C (T3) reaching values of about 6 log cell/g (with both quantification methods). After 7 days, control cells reached up to 7 log cell/g according to qPCR, while the culturability remained comparable to T3. Concerning heat treated cells, only a slight increase of the culturability was observed at T3 and T7, while the total number of cells detected by qPCR remained constant. Analogously, the total concentration of the heat treated cells in presence of terpens remained constant, while some of the cells reacquired their culturability after 3 (T3) and 7 (T7) days at 4 °C (Fig. 4B), reaching up to 3 log CFU/g. These results suggest that the increased values observed could be due to a resuscitation of VBNC cells rather than the cell ability to duplicate on Gorgonzola rind.

Differences among the two matrices can be explained by the competition between spiked cells of *L. monocytogenes* and the microorganisms naturally present on foods. This aspect was already underlined by Broady et al.^33^ which observed the inability of L*. monocytogenes* to grow, even if inoculated, in some roast beef samples. McDonnell et al.^35^ observed a lower growth of this pathogen in roast beef if compared with other meat product, and a 2 log cfu/g cell increase required 4 weeks at 4 °C. On the other side, blue veined cheeses such as Gorgonzola, are favorable substrates for *L. monocytogenes*^32,36^ and lactic acid bacteria, which dominate the microbiota, show limited anti-listerial activity, especially if the contamination takes places after the ripening period^37^.

## Conclusions

*L. monocytogenes* is a concern in the food industry and several methods to prevent its replication on foods are being investigated. Among them, the combination of mild heat treatment and natural antimicrobials as terpenoids thymol and carvacrol resulted in a synergistic effect for a partial or total inactivation of *L. monocytogenes*^6^. However, the connection between the inoculation method and the effectiveness of the antimicrobial treatment has never been explored in this foodborne pathogen. Moreover, the ability of the viable but not culturable cells to recover their ability to replicate remains a dark point which needs clarification.

In our study we highlighted for the first time a link between the origin of the cells and the sensitivity towards an antimicrobial treatment in *L. monocytogenes*. Here, we found that cells from inoculation method 1 (from colony to BHI broth) resulted less sensitive to the thermal treatment combined with terpenoids. This finding could indicate that the physiological state of the cells is a determinant for effectiveness of the treatment. Indeed, both culturability and viability were differently affected by the antimicrobial treatment depending on the inoculation method. The effectiveness of a treatment is in general assessed by standard laboratory culture techniques, that do not allow the detection of VBNC cells. This state is induced by environmental stress (pH, temperature, thermal stress, antimicrobials)^27^. Therefore, the common laboratory practice may lead to an overestimation of the efficacy of the treatment. In this context, flow cytometry represents a good strategy that can be integrated with classical plate counting, because it allows to reveal not only viable cells, but injured and dead cells as well. The VBNC population, for definition, is non culturable and it can represent a risk for producer and consumer. Indeed, this part of the population can resuscitate under favorable conditions and recover the ability to duplicate. In our study, the recovery ability was assessed both in vitro (in BHI broth) and in vivo (Gorgonzola rind and roast beef). Although only preliminary results, our data confirmed that heat treated cells were able to partial recover their culturability in synthetic medium and when inoculated on Gorgonzola rind as well, likely due to a higher availability of nutrients and other factors intrinsic to this food matrix (pH, water activity, relative humidity).

In conclusion, our results stressed the importance of the cells history in the effectiveness of the antimicrobial treatment and the relevance to determine a common protocol for antimicrobial sensitivity assessment, to obtain comparable results among different laboratories. Although the reason of this effect needs further investigations, differences in cell history can strongly impact the outcomes of the assays, therefore raising safety concerns for health’s consumer.

## Materials and methods

### Bacterial strains and culture conditions

The strain used in this study was *L. monocytogenes* Scott A belonging to the collection of the Department of Agricultural and Food Sciences (University of Bologna). The strain was maintained as glycerol (20% v/v) stock at − 80 °C (condition 2, Fig. 1). The strain was pre-cultured in Brain Heart Infusion (BHI; Oxoid, UK) for 24 h at 37 °C at 200 rpm-shaking conditions (Heidolph, Germany). Part of these cells were then used to inoculate (i) a BHI agar plate to obtain a single colony for a new flask inoculation (conditions 1 and 3, Fig. 1) and (ii) a new flask of BHI (condition 4, Fig. 1). Cold adapted cells of condition 3 were kept at 4 °C in a flak. Cells from condition 1 to 4 were used to investigate the effect of the cell origin on the response of this strains to thermal treatment combined or not with terpenoids (thymol and carvacrol). Each condition was tested in triplicate.

### Effect of thermal treatments and aroma compounds on the viability of *L. monocytogenes*

Cells deriving from the different pre-cultures were resuspended at a cell load of approx. 6 log viable cells/ml (or Active Fluorescent Unit, AFU, determined by flow cytometry, see “Flow cytometric analysis”) in sterile PBS (NaCl 9 g/l, Na_2_HPO_4_ 0.421 g/l, KH_2_PO_4_ 0.144 g/l, pH 7.4), pre-heated at 55 °C in a water bath LAUDA Ecoline (LAUDA-Brinkmann, LP., Delran, New Jersey, US). Isothermal treatments at 55 °C for 30 min were performed in 50 ml sterile tubes with or without the addition of terpenes. Particularly thymol and carvacrol (both provided by Sigma-Aldrich, St. Louis, MO) were previously dissolved in ethanol and then added just before inoculum at a concentration of 50 mg/l each. These concentrations were chosen based on the results obtained in a previous study^6^ and were below the MIC values (20 and 25% of MIC for thymol and carvacrol, respectively) to not significantly affect per se cell culturability and viability. As control, we used a cell suspension treated with 0.5% (v/v) ethanol^6^.

Samples were collected at the end of the different treatments to evaluate: (i) cell culturability through plate counting onto BHI agar, by diluting cells suspension in PBS (pH 7.4), and incubation at 37 °C for 24 to 48 h; (ii) cell viability through flow cytometry (see “Flow cytometric analysis”); (iii) cell recovery in BHI broth at 37 °C. For this latter, the treated cell suspensions were inoculated 1:100 in fresh BHI in a 96 well plates (28 wells for each condition) through an automatic liquid handling system (EpMotion, Eppendorf, Italy) in a final volume of 200 μl/well. Then microplates were incubated at 37 °C for 48 h and cell growth was monitored using a spectrophotometer EON (Biotek, Winoosky, VT) programmed for readings (OD 600 nm) every 15 min.

### Flow cytometry analysis

Cell suspensions collected after each treatment were analysed by flow cytometry using an Accuri C6 Plus flow cytometer (BD Biosciences, Milan, Italy). Setting parameters, emission filters and thresholds were defined according to Arioli et al.^6^. The cell suspensions were stained in the dark for 15 min at 37 °C with 1 µM SYTO 24™ (ThermoFisher Scientific, Milan, Italy) and 2 µM Propidium Iodide (Merck, Italy). SYTO 24™ permeates the membrane of total cells and stains the nucleic acids with green fluorescence; PI penetrates only bacteria with damaged membranes, causing a reduction in SYTO 24™ green fluorescence when both dyes are present. This dual staining allowed to distinguish three sub-populations: Active Fluorescent Unit (AFU, considered as alive cells), injured cells and non-Active Fluorescent Unit (non-AFU, considered as dead cells)^38^.

### Growth modeling

The data obtained from cell recovery, in which *L. monocytogenes* cells after the different treatments were inoculated in fresh BHI medium (initial concentration 4 log cfu/ml) and monitored for 48 h through the variation of optical density at 600 nm (OD_600_), were modeled with Gompertz equation as modified by Zwietering et al.^28^.

$$y=A{e}^{{-e}^{\left[\left(\frac{{\mu }_{max}e}{A}\right)\left(\lambda -t\right)+1\right]}}$$

where *y* is the OD_600_ at time t, *A* represents the maximum OD_600_ value reached, *µ*_*max*_ is the maximum OD_600_ increase rate and *λ* is the lag time.

### *L. monocytogenes* cell recovery evaluation in food systems

The recovery ability of treated *L. monocytogenes* cells on food system was assessed by qPCR and by standard dilution and plating. The selected matrices were Gorgonzola cheese and roast beef, bought in a local supermarket. The cheese was sold as a slice contained in a hard-plastic packaging while the roast beef was thinly sliced and sold over the counter and is a common ready-to-eat meat in Italy. For the total quantification by a culture-independent method, standard curves were constructed from serially diluted cells of *L. monocytogenes* inoculated onto the food matrix. Gorgonzola cheese rind and roast beef slices were cut into pieces of 1 g, then placed in a sterile Petri dish where they were inoculated on their surface with 50 μl of cell suspension containing from 10^4^ up to 10^7^ cells/g. After the liquid had been adsorbed, we proceeded with the DNA extraction protocol (see “DNA extraction from food samples and qPCR analysis”) and subsequent qPCR analysis. Standard curve was constructed by plotting the threshold cycle (C_T_) values obtained against the log number cells. The data obtained were expressed as cell number per gram of food matrix. The spiking of a food matrix with treated cells suspensions was carried out as follows. An overnight culture of *L. monocytogenes* was prepared and used in thermal treatment in presence or not of terpenoids (see protocol in “Effect of thermal treatments and aroma compounds on the viability of *L. monocytogenes*”). The treated cell suspensions were then used to spike Gorgonzola rind cube or roast beef strips that had been prepared in the same manner as the ones used to establish the standard curves. The suspensions used were: (i) non-treated cells (positive control), (ii) thermally treated cells at 55 °C for 30 min; (iii) thermally treated cells at 55 °C for 30 min in presence of 50 mg/l of each terpenoid, (iv) only PBS (as negative control). After inoculation of the food matrix, samples were stored at 4 °C for 3 and 7 days. All the samples were subjected to analysis for total and culturable *L. monocytogenes* load determination on ALOA agar medium (Biolife, Italy). The analyses were performed in triplicate.

### DNA extraction from food samples and qPCR analysis

The DNA from food matrixes (250 mg) was extracted by using DNeasy® PowerLyzer® PowerSoil® (Qiagen, Italy) according to manufacturer’s protocol. Mechanical lysis of cells was carried out using a bead beater (Precellys 24, Bertin Technologies, Montigny le Bretonneux, France). The DNA extracted from food samples was quantified by using a NanoDrop (BioTek Instruments, Inc., CA, United States). Finally, the DNA was stored at – 80 °C until molecular analysis. Real-time quantitative PCR (qPCR) protocols were adopted for the quantification of *L. monocytogenes* in food metagenomic DNA, targeting bile salt hydrolases gene *bsh*, bshF: GGCCTTAGTATGGCAGGACTCA and bshR: CTCATTGTCCTTACCTTCTGCAAA^39^.

The amplification reaction was carried out in a final volume of 15 ml containing 7.5 ml of EvaGreen.

R Supermix (Bio-Rad Laboratories, Segrate, Italy) and 0.5 mM of each primer; 50 ng of template DNA samples was used in each reaction. The amplification was carried out using the following thermal program: 95 °C/3 min; (95 °C/10 s, 63 °C/30 s, 72 °C/5 s) × 39 times. The standard curve was obtained by plotting the average Cq values versus log10 of the number of cells added to each food sample. Melting curves were analysed with Bio-Rad CFX Manager 3.1 software to confirm the specificity of the amplification products.

The cell culturability was assessed by diluting 1 g-Gorgonzola rind or roast beef slice in PBS (pH 7.4) and plating on ALOA (Biolife, Milan, Italy) for the discrimination and counting of *L. monocytogenes* cells.

### Statistical analysis

To assess the sensitivity of *L. monocytogenes* to thermal treatments and aroma compounds, three independent experiments were carried out. The data were statistically analysed using the one-way ANOVA. The Tukey critical difference test was performed to determine differences among different conditions (p < 0.05). The parameters of the Gompertz equation were estimated by non-linear regression. The presence of significative differences among the λ estimates was evaluated by using the non-parametric Kruskal Wallis test (p < 0.01). Statistica for Windows 6.1 package was used for analyses (Statsoft Italia, Vigonza, Italy).
