# Author Correction: *Listeria monocytogenes* sensitivity to antimicrobial treatments depends on cell origin

**DOI:** 10.1038/s41598-022-07641-2

**Published:** 2022-03-01

**Authors:** Chiara Montanari, Giulia Tabanelli, Federica Barbieri, Diego Mora, Robin Duncan, Fausto Gardini, Stefania Arioli

**Affiliations:** 1 Department of Agricultural and Food Sciences, University of Bologna, Bologna, Italy; 2 Interdepartmental Center for Industrial Agri-Food Research, University of Bologna, Bologna, Italy; 3 Department of Food, Environmental and Nutritional Sciences (DeFENS), Università Degli Studi di Milano, Via Celoria 2, 20133 Milan, Italy

Correction to: *Scientific Reports* 10.1038/s41598-021-00767-9, published online 28 October 2021

The original version of this Article contained errors in the spelling of the authors Chiara Montanari, Giulia Tabanelli, Federica Barbieri, Diego Mora, Robin Duncan, Fausto Gardini & Stefania Arioli, which were incorrectly given as Montanari Chiara, Tabanelli Giulia, Barbieri Federica, Mora Diego, Duncan Robin, Gardini Fausto & Arioli Stefania.

The original Article has been corrected.

